# Fabricating fast triggered electro-active shape memory graphite/silver nanowires/epoxy resin composite from polymer template

**DOI:** 10.1038/s41598-017-05968-9

**Published:** 2017-07-17

**Authors:** Jie Zhou, Hua Li, Ran Tian, Roberto Dugnani, Huiyuan Lu, Yujie Chen, Yiping Guo, Huanan Duan, Hezhou Liu

**Affiliations:** 1 State Key Laboratory of Metal Matrix Composites, School of Materials Science and Engineering, Shanghai Jiao Tong University, Shanghai, China; 2 Collaborative Innovation Center for Advanced Ship and Deep-Sea Exploration, Shanghai Jiao Tong University, Shanghai, China; 3 University of Michigan-Shanghai Jiao Tong University Joint Institute, Shanghai, China

## Abstract

In recent years shape-memory polymers have been under intense investigation due to their unique mechanical, thermal, and electrical properties that could potentially make them extremely valuable in numerous engineering applications. In this manuscript, we report a polymer-template-assisted assembly manufacturing strategy used to fabricate graphite/silver nanowires/epoxy resin (PGSE) composite. In the proposed method, the porous polymer foams work as the skeleton by forming three-dimensional graphite structure, whereas the silver nanowires act as the continuous conductive network. Preliminary testing on hybrid foams after vacuum infusion showed high electrical conductivity and excellent thermal stability. Furthermore, the composites were found to recover their original shape within 60 seconds from the application of a 0.8 V mm^−1^ electric field. Notably, the reported shape-memory polymer composites are manufactured with readily-available raw materials, they are fast to manufacture, and are shape-controlled.

## Introduction

Shape-memory polymers (SMPs) and their composites (SMPCs), can recover their original (or permanent) shape upon exposure to external stimuli^1^. There are various types of methods to stimulate the deformation, of which thermal- and electro-responsive are the most common^2, 3^. SMPs have numerous advantages such as being lightweight, inexpensive, and easy to manufacture; additionally they display high deformability, good biodegradability and easy-to-tailor glass transition temperatures compared with shape memory alloys and shape memory ceramics^4–7^. Therefore, in recent years an increasing number of international researchers have been focusing on the development of the shape memory effect in polymers^8, 9^. Comparing with SMPs, SMPCs have higher strength, higher stiffness, and distinctive physical characteristics determined by the type and amount of added fillers. So many attractive features have already inspired a variety of applications in the fields of aerospace^10^, biomedicine^11^, textiles^12^, etc.

Graphite, which has outstanding electrical^13^, mechanical^14^, and optical properties^15^, has been receiving considerable attention as one of the most promising engineering materials. However, graphite’s poor dispersion characteristics have partially limited its application^16^. Various methods have been proposed to improve graphite’s dispersion, such as for instance chemical exfoliation^17^. Unfortunately, graphite oxide synthesized by chemical exfoliation has been found to display abundant defects and chemical moieties^18, 19^, which significantly decrease its electrical conductivity. Even after graphite oxide (GO) is reduced, the reduced graphite oxide (RGO) is still less conductive than pristine graphite^20^. In addition, aggregation and stacking between individual graphite sheets (driven by the strong π-π interaction) greatly compromise the intrinsically high specific surface area of graphite. Furthermore, the high conductivity of graphite is also largely compromised by the inter-sheet contact resistance. The three-dimensional (3D) graphite structure was deemed to alleviate the aforementioned problems currently hindering the performance of graphite composites. The 3D graphite network is continuous and displays higher conductivity due to increasing inter-sheet junctions. It is believed that developing 3D structures of graphite would further expand its range of applications. Several approaches have been developed for the fabrication of 3D interconnected structures such as freeze casting^21^, self-gelation^22^, and chemical vapor deposition (CVD)^23^. However, these 3D graphene structures were reported to either fail in a brittle manner or to undergo significant plastic deformation when cyclically loaded^24^. For example, Zhou and co-workers proposed synthesizing the carbon nanotube sponge shape-memory polymer nanocomposite using CVD, and showed that the composite could be triggered within 10 s from the application of 10 volts^25^. Nonetheless, the deposition rate of CVD is generally not high, and in many manufacturing methods, the reaction source and the residual gas are either flammable or toxic. Furthermore, the sample dimensions manufactured with this technique are smaller than the ones produced with the proposed method, and the deformed angles are less than 90°. Lu and co-workers have reported that by adding SMPs coating of carbon paper on the SMPs surface and by further adding carbon nanofibers, the sample could recover its original shape within 180 seconds^26^. Elsewhere, Lu and co-workers also concluded that the electrical actuation of SMP composites coated with 1.8 g carbon nanofiber nano-paper could recover the original shape within 140 s^27^. Nonetheless, for these composite materials, the deformed shapes had to be less than 180° otherwise the shape recovery rate was found to be slower than the one proposed in this work.

**Figure 1:**
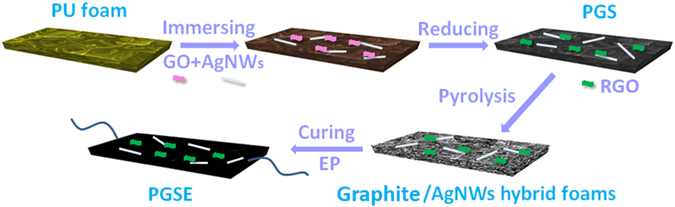
Overall fabrication process of the PGSE composites, including: 1) Immersion of a commercial PU foam into the mixture of AgNWs, GO, and Vitamin C; 2) Assembly of graphite and AgNWs on the PU skeleton by *in-situ* chemical reduction of GO; 3) Pyrolysis of the PGS to obtain graphite/AgNWs hybrid foams; 4) Immersion of graphite/AgNWs hybrid foams into the epoxy resin and curing.

In this work, we employed a simple yet effective polymer-template-assisted assembly strategy for fabricating 3D graphite/silver nanowires foam (PGS). The polymer templates are easily removed by pyrolysis, in which charge carriers can pass through the defect region of graphite rapidly with the aid of high quality Ag nanowires (AgNWs) building blocks. Preliminary experiments indicate that the resulting 3D network shows high electrical conductivity. In this work, the shape memory composite was prepared by a vacuum infusion method. The synthesis process is shown schematically in Fig. 1. The proposed PGSE composites demonstrated excellent conductivity and could be triggered within 60 seconds by applying an electric field of approximately 0.8 V mm^−1^ across it. The composites manufactured with the proposed method could be triggered with a lower voltage and could recover their original shape in shorter time, in addition to displaying high shape-fixity ratio and shape-recovery ratio. In the future, the shape memory composites presented in this work are likely to be a suitable candidate for the large-scale production for applications in the aerospace field and adaptive optical devices among the others.

## Results and Discussion

**Figure 2:**
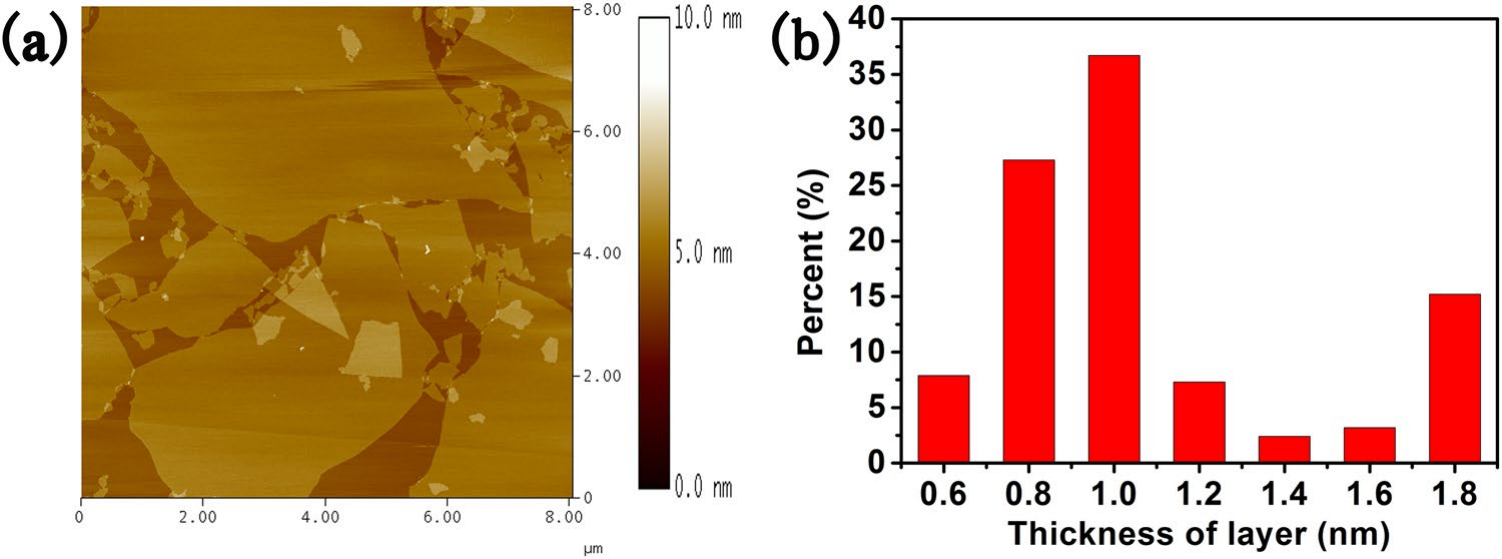
(**a**) AFM image of exfoliated GO sheet. (**b**) Statistical results of GO sheet thickness.

**Figure 3:**
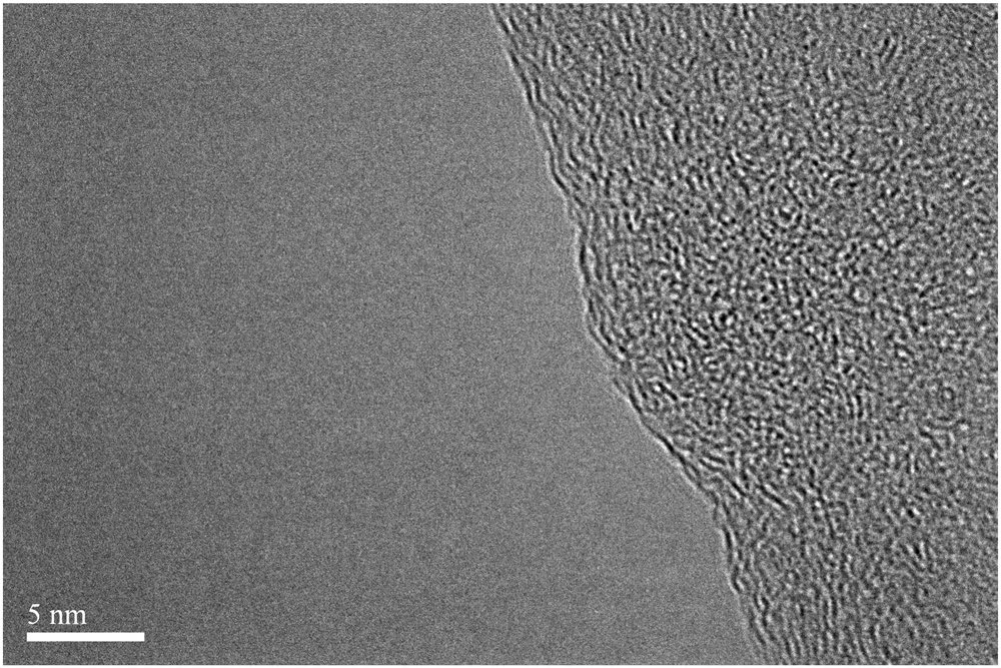
HRTEM image of thin GO sheet.

The aqueous solution of GO sheets was dropped onto mica substrate. The thickness of suspended GO sheets were characterized by atomic force microscope (AFM) and the statistical results are shown in Fig. 2. Most of GO sheets are less or around 1 nm, suggesting that thin oxidized graphite was achieved. Different from chemical vapor deposition method^28, 29^, the monolayer GO achieved from Hummers method has irregular edges^30–33^ (see Fig. 3).

**Figure 4:**
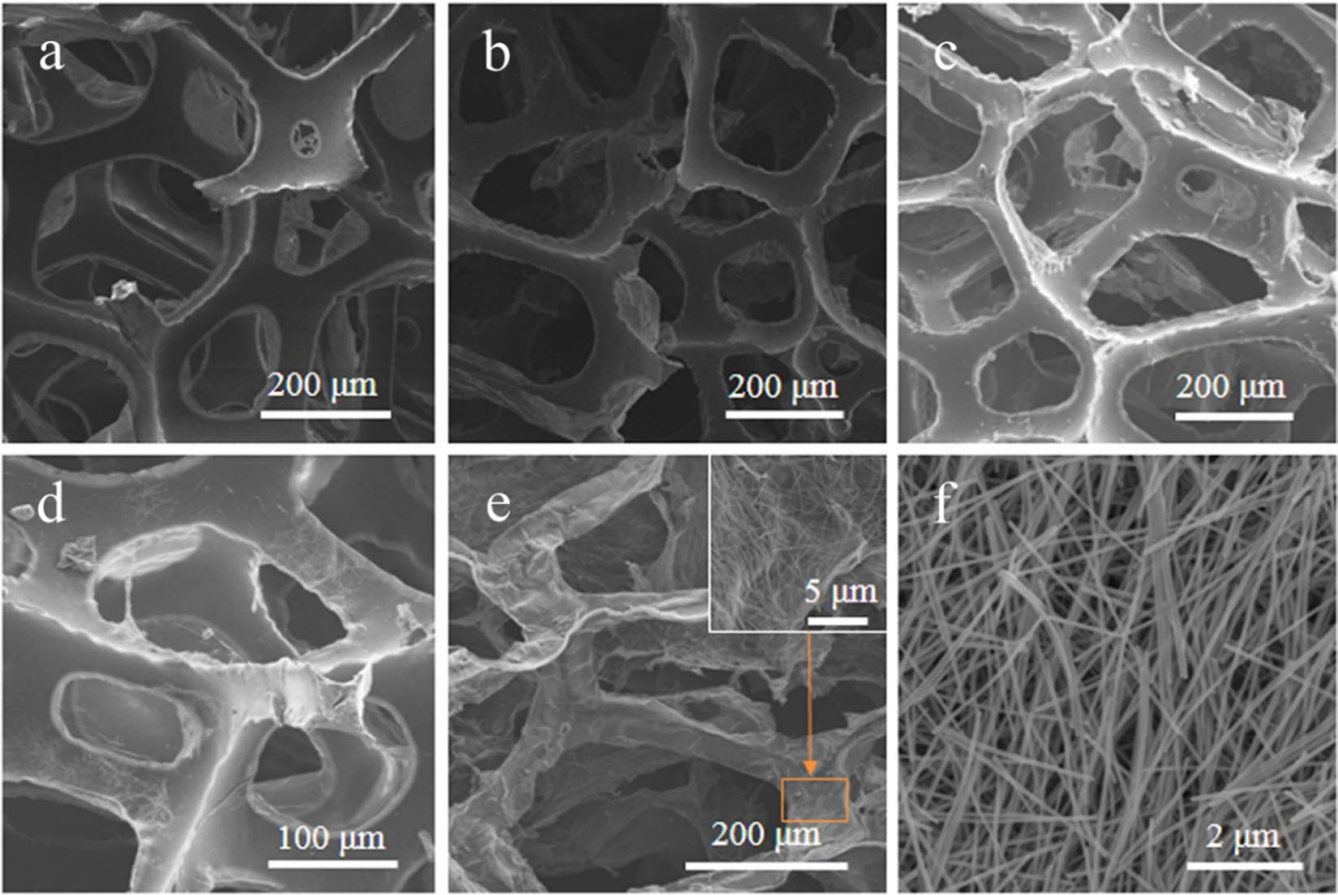
(**a**) SEM image of PU foam. (**b**) PG. (**c**) PU based graphite/AgNWs (1:1) hybrid foam. (**d**) PU based AgNWs foam. (**e**) PU based graphite/AgNWs (1:1) hybrid foam after removal of polymer-templates. (**f**) SEM image of AgNWs.

**Figure 5:**
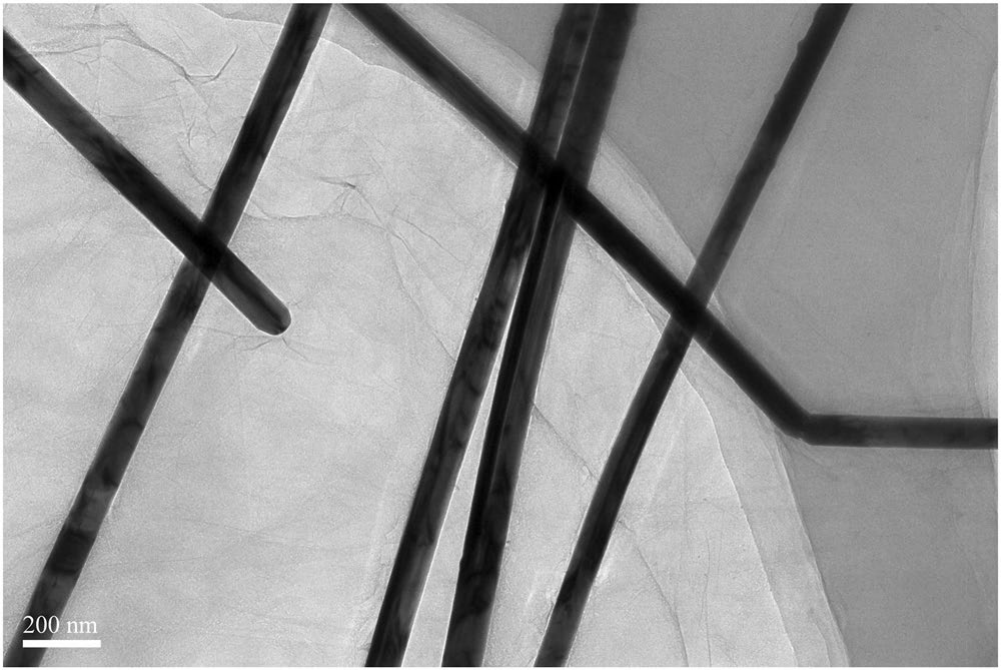
TEM image of the mixed solution with a volume ratio of graphite/AgNWs (1:1).

Figure 4a shows the SEM image of a PU foam. It can be observed that the PU foam displayed a porous 3D network structure with macro-pores of hundreds of micrometers in diameter. PG and PGSs displayed similar structure as the PU foam, while the hybrid foams containing graphite exhibited a crinkled and rough texture. This phenomenon suggested that the graphite sheets were fixed around the PU skeleton during the reducing process. Figure 4e shows the hybrid foams after removal of polymer-templates. It still displays interconnected, porous 3D structure. The residual components are graphite sheets and AgNWs. Figure 4e shows a magnified image of the sample with graphite and AgNWs homogeneously dispersed in the composites. This could also be observed in the TEM image of the same sample (Fig. 5).

**Figure 6:**
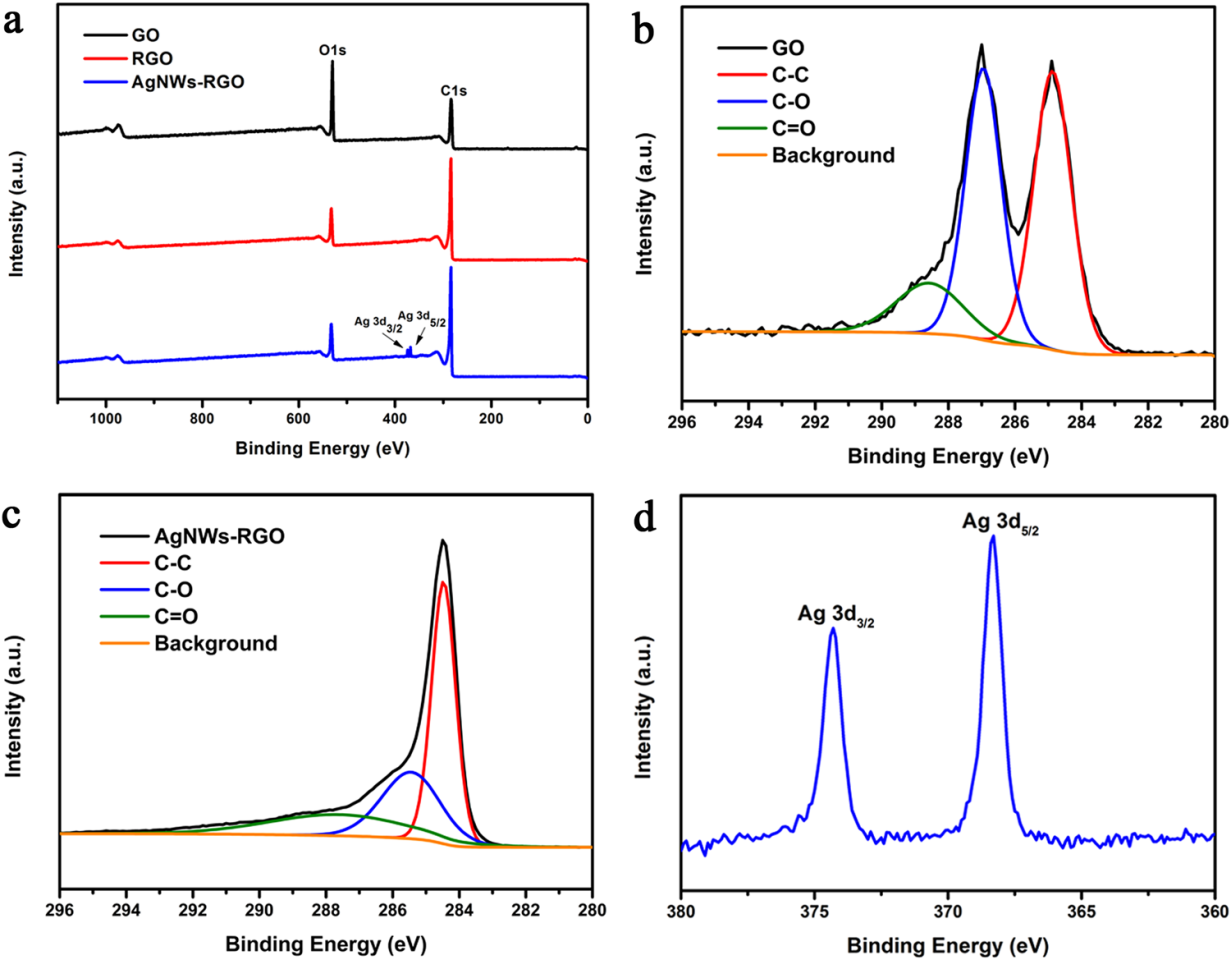
(**a**) XPS spectra of pristine GO and RGO sheets stripped from the PG, and RGO/AgNWs (1:1). (**b**) C 1s spectra of GO. (**c**) C 1s spectra of RGO/AgNWs (1:1). (**d**) Ag 3d spectra of RGO/AgNWs (1:1).

The GO, RGO, AgNWs/RGO composites were further characterized by XPS spectrum. Typically, the C1s and O1s peak of GO and RGO could be observed at 284 eV and 530 eV. In order to conduct the quantitative analysis, both of the C1s and O1s spectra need to be separated into their individual components. The XPS spectrum of GO shows the three main types of C1s peaks at 284.6 eV, 286.6 eV and 287.8 eV (Fig. 6b), which were assigned to C–C, C–O and C=O, respectively^34^. The intensities of all C1s peaks from AgNWs-RGO composites decrease dramatically (Fig. 6c), indicating that the oxygenated functional carbon groups on GO were removed after reducing; the C/O atomic radio of GO was 70/30, whereas after reducing by Vitamin C, the C/O atomic ratio of AgNWs-RGO was 93/7. The concentration obtained from XPS for the PGSE composite was 90 C atom%, 7 O atom%, and 3 Ag atom%. The mass concentration of C/Ag was 3.3:1. In the AgNWs-RGO spectrum, significant Ag3d signals appeared (Fig. 6d).

**Figure 7:**
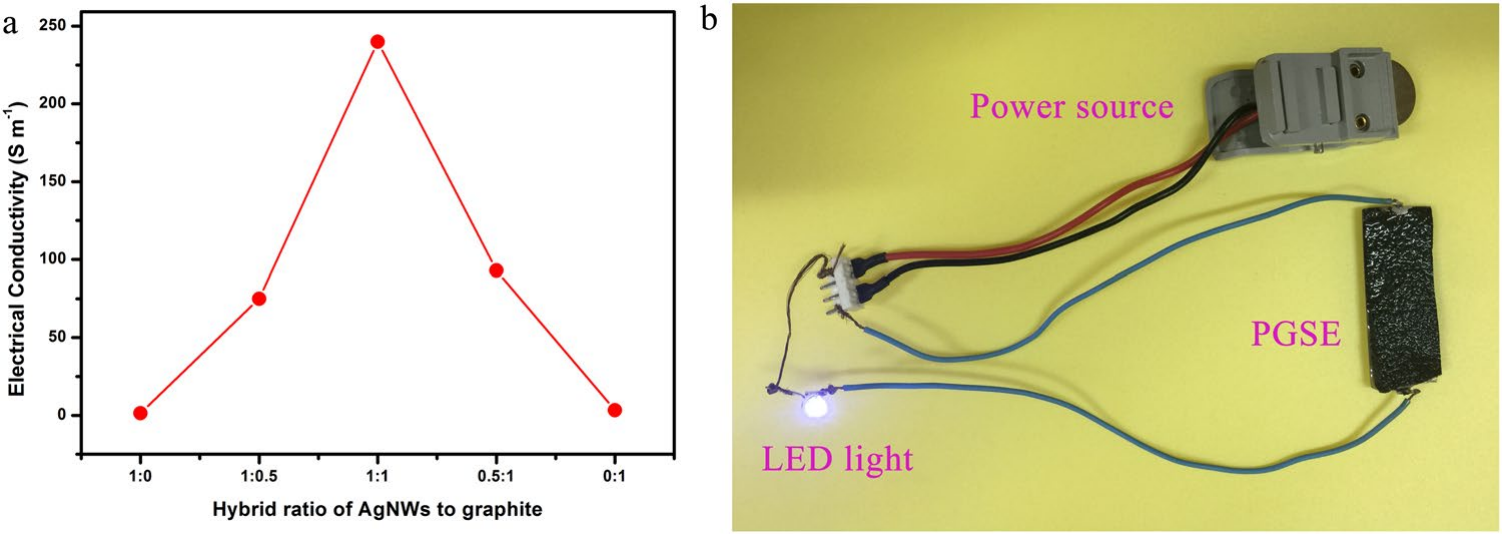
(**a**) Electrical conductivity variation of the PGS with a volume ratio of AgNWs/graphite (1:0, 1:0.5, 1:1, 0.5:1, 0:1). (**b**) Optical image of the LED light when connecting the PGSE composites with 10 wt% graphite sheets and 3 wt% AgNWs in the circuit.

The electrical conductivity of PGSs was measured by the four-point probe method. The results are shown in Fig. 7a. The hybrid foam shows low density and high electric conductivity. The PGS with a volume ratio of AgNWs to graphite (1:1) for example, displays density of about 20 mg cm^−3^. The electrical conductivity is about 240 S m^−1^, which is higher than the value of PU foam with only graphite (3.3 S m^−1^) and PU foam with only AgNWs (1.4 S m^−1^), and it is higher than PGS with a volume ratio of AgNWs to graphite (2:1, 75 S m^−1^), and also higher than PGS with a volume ratio of AgNWs to graphite (1: 2, 93 S m^−1^). Such outstanding conductive performance could probably be attributed to two main factors: first, the 1D AgNWs bridge defects in the 2D flexible graphite sheets and work as nanowires; second, the AgNWs adhering to graphite sheets act as ribs preventing the agglomeration of the graphite sheets and decrease the interlayer contact resistance. However, the fragile structure of the compound aerogel is very sensitive to the vacuum infusion process and its structure is partially damaged, hence the conductivity of the composites decreased^35^. On the other hand, the polymer template has much lower thermal decomposition temperature in comparison with graphite and AgNWs, and it could be easily removed in the pyrolysis process. In addition, the reduction level of GO and the interfacial junction between graphite and AgNWs could be enhanced^36^. Considering the above two factors, combined with experimental results, changes in conductivity could be ignored before and after the curing process.

**Figure 8:**
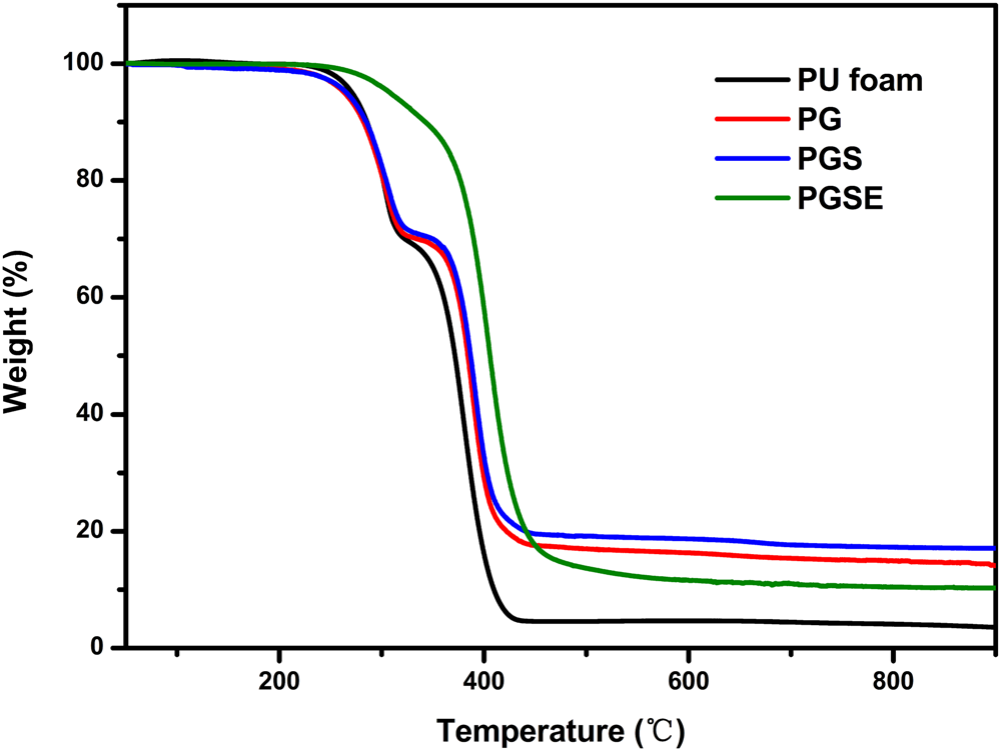
TGA curves of PU foam, PG with 10 wt% graphite sheets, PGS with 10 wt% graphite sheets, 3 wt% AgNWs, and PGSE composite with 10 wt% graphite sheets and 3 wt% AgNWs, respectively.

**Figure 9:**
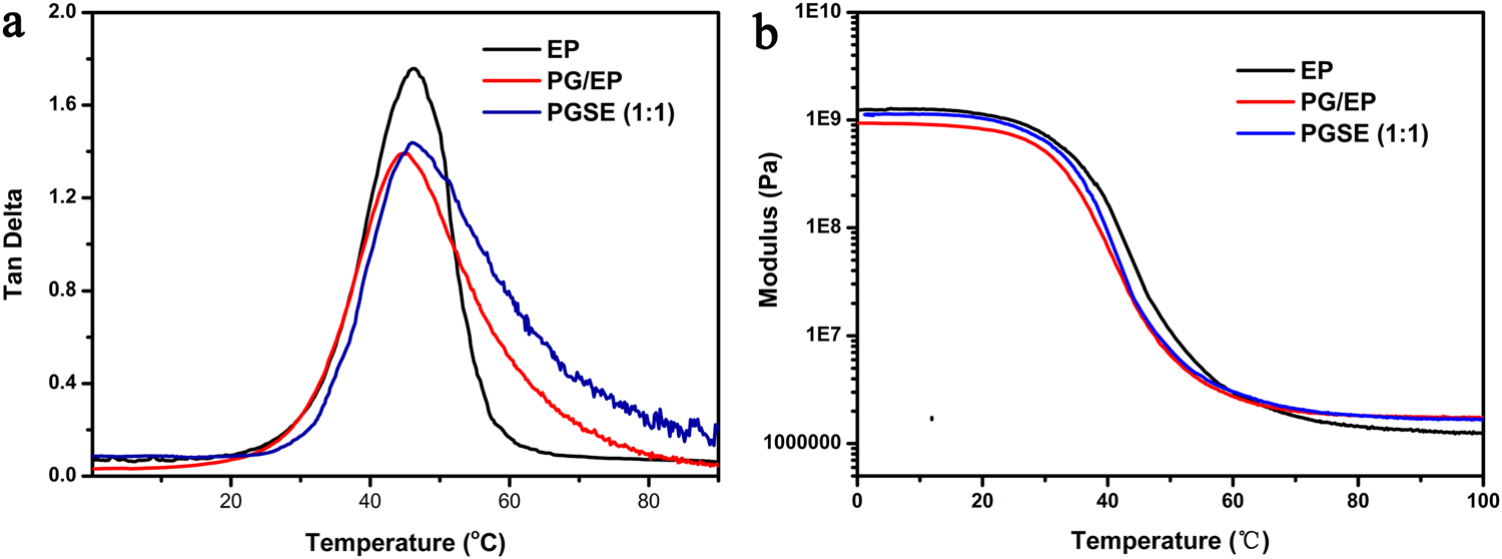
Dynamic mechanical properties of pristine EP, PG/EP with 10 wt% graphite sheets, and PGSE with 10 wt% graphite sheets and 3 wt% AgNWs, respectively: (**a**) tan delta, (**b**) storage modulus.

TGA was used to assess the thermal degradation temperatures of pure PU foams, PU-based graphite foams (PG), PGS with a volume ratio of AgNWs to graphite (1:1), and PGSE composite with a volume ratio of AgNWs to graphite (1:1). Before the addition of epoxy resin (EP), all materials showed a two-stage degradation: an early stage starting at around 250 °C, with a maximum rate at about 300 °C, assigned to the urethane bond breaking^37^. The PGSE composite displayed a total 88 wt% at 550 °C and no obvious additional weight loss was detected above 550 °C. It can be seen that the composite containing EP showed higher thermal stability. The reason could be ascribed to the fact that the thermal stability of EP is higher than that of PU. In addition, the grafting between –NCO of PU and the –OH of EP was also beneficial to the thermal stability^38, 39^. Comparing the four traces in Fig. 8, the final char of each of the pure materials was used to calculate the approximate concentration of each component. Considering the char results obtained for the composite^40^, it was estimated that approximately 10 wt% for graphite and 3 wt% for silver nanowires were present in the PGSE composite, which agreed well with the XPS results. Dynamic mechanical analysis was operated to evaluate the viscoelastic properties of the cured composites. The analysis considered pristine EP, PG/EP, and PGSE (1:1), respectively. The glass transition temperature (*T*_g_) values were assumed as the temperatures corresponding to maxima in the tan delta curves^41^. As is shown in Fig. 9a, epoxy resin, graphite, and silver nanowires displayed a good compatibility, since each tan delta-temperature curve has only one peak. The pristine EP was found to have a *T*_g_ of 48.0 °C. The addition of graphite, led to non-stoichiometric balance between the epoxy resin and the curing agents, resulting in the reduction of the cross-linking density and hence decreasing the *T*_g_ value (*T*_g_ of PG/EP is 43.9 °C)^35^. Conversely the addition of AgNWs decreased the mobility of polymer chains in the matrix, resulting in the increase of *T*_g_ to 45.9 °C^42^.

The storage modulus of pristine EP, PG/EP, and PGSE (1:1) are shown in Fig. 9b. The incorporation of nano-fillers into a polymer matrix could influence the storage modulus through the interfacial bonding strength change between reinforcement filler and resin matrix^43^. When the temperature was below the *T*_g_, the modulus for the composite samples containing graphite and AgNWs was lower than their corresponding matrices, which is probably caused by the weakened cross-linking density. As the temperature increased, the storage modulus sharply decreased as the result of the energy dissipation during the transition from the glassy state to the rubber state. Comparing the storage modulus in the rubbery region, the storage modulus of every composite is higher than that of the corresponding matrices, which probably could be attributed to the confinement effect of graphite and AgNWs^44^.

**Figure 10:**
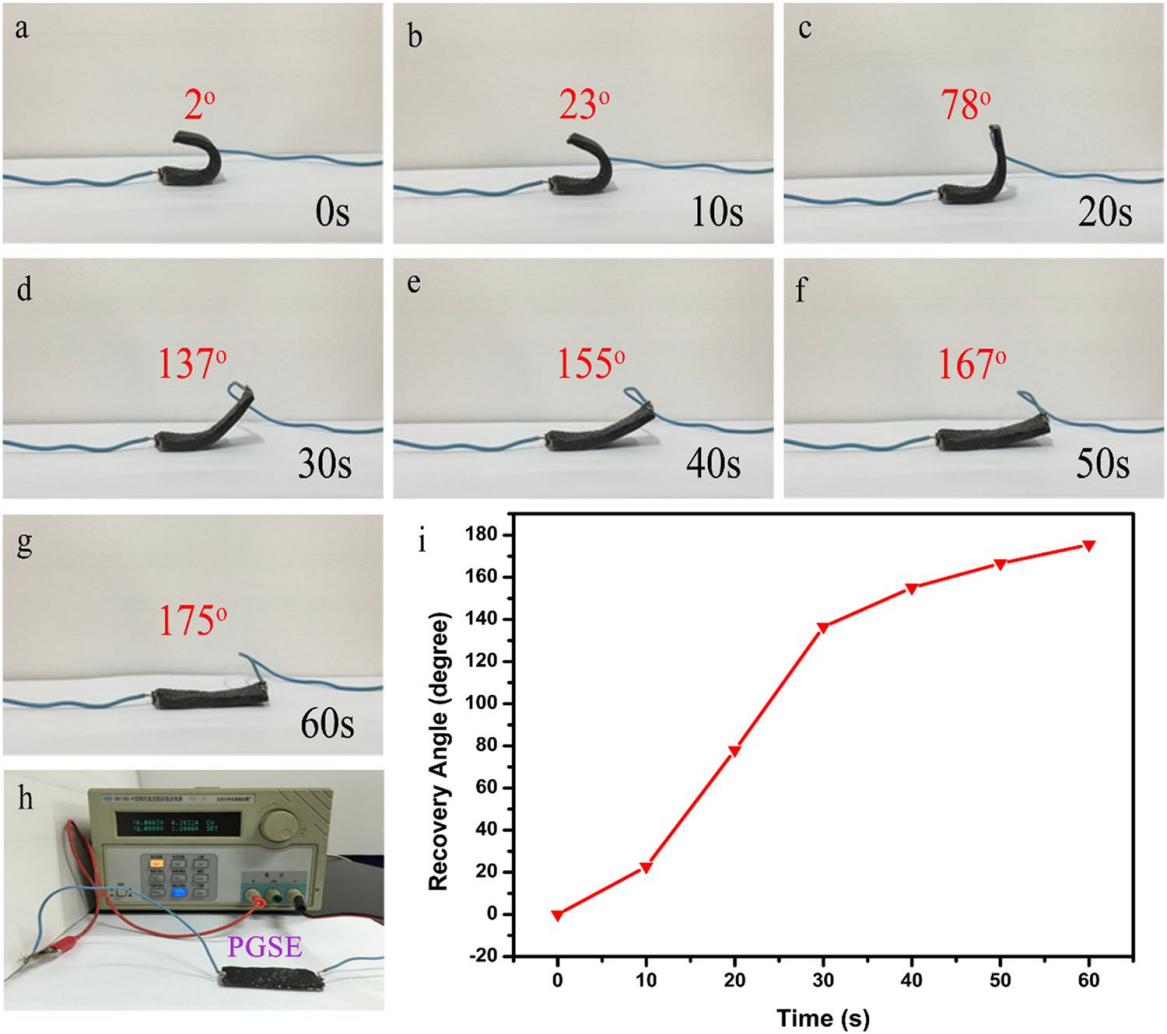
(**a**–**g**) Shape recovery process of the PGSE composite with 10 wt% graphite sheets and 3 wt% AgNWs under voltage of 40 volts. (**h**) Optical image of the circuit of the shape memory process. (**i**) The relationship of recovery time and recovery angle.

Because of the continuous 3D graphite and AgNWs electrical conductive network, when connected to a power source with a voltage of 40 volts the PGSE could recover its original shape without the use of an external heat source. The shape recovery was accomplished by letting the internal resistance of the composite heat up the sample. Shape recovery testing was conducted on one PGSE composite with 10 wt% graphite sheets and 3 wt% AgNWs. The PGSE tested consisted of a rectangular beam sample of dimensions of 50 mm × 20 mm × 4 mm. The shape recovery process is also shown in Fig. 10.

Before the shape recovery process, the following steps were taken: first, the sample was heated to 70 °C at an approximate rate of 5 °C min^−1^ and kept at 70 °C for 10 min to ensure a uniform temperature distribution. Then the sample was bent around a preheated PTFE cylinder with a diameter of 10 mm, to form an angle of 180°. The bent samples were fixed on the mandrel, immersed in the cool water, and held for 10 min to ensure the samples retained the imparted U shape. The final bending angle, θ_1_, was then recorded. The PGSE was connected to a DC power with copper wires fixed at each end of the sample. By applying a voltage of 40 volts, the PGSE nearly recovered its original shape within 60 s. It was found that during the first 10 s, the recovery rate was only 2.3 degrees per second; subsequently, the recovery rate gradually increased up to 5.7 degrees per second; finally, in the last 20 seconds, the recovery rate decreased to 1 degree per second since a large proportion of the stress had been released in the earlier stages. The final angle was recorded as θ_2_. The shape fixity ratio (*R*_f_) and shape recovery ratio (*R*r) were calculated using Eqs. (1) and (2).

1 $${R}_{f}=\frac{{{\rm{\theta }}}_{1}}{180}\times 100 \% \,$$

2 $${R}_{r}=\frac{180-{{\rm{\theta }}}_{2}}{180}\times 100 \% $$

**Figure 11:**
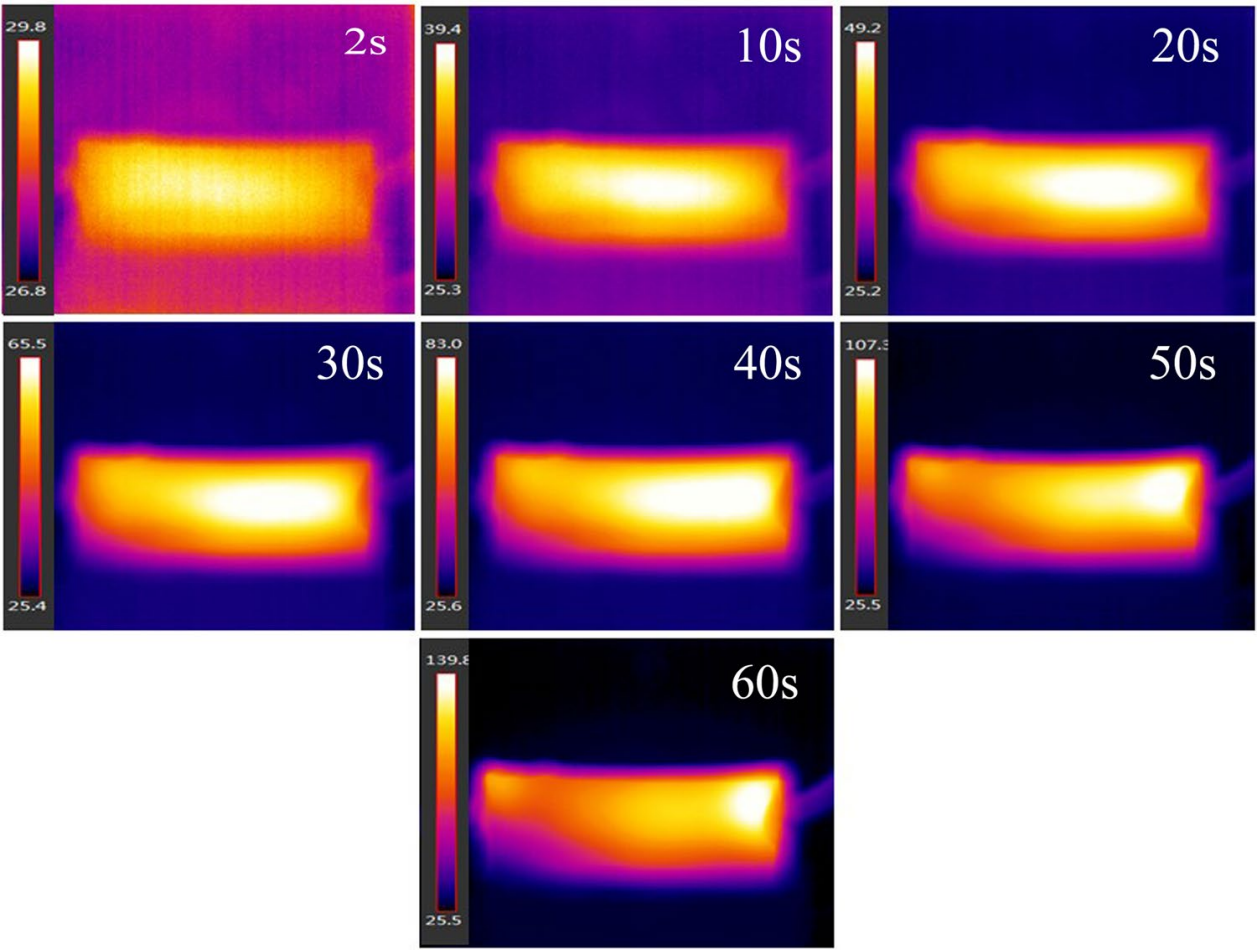
Surface temperature distribution of the PGSE with 10 wt% graphite sheets and 3 wt% AgNWs in the recovery process.

In our experiment, *R*_f_ and *R*_r_ values were nearly 99% and 97%. The surface temperature distribution in the process of electrical triggering of the composite was characterized using an infrared radiation thermometer. As is illustrated in Fig. 11, the majority of the sample’s surface exceeded the *T*_g_ within 20 s from actuation, implying that the inner 3D network of graphite and AgNWs could generate enough heat to guarantee the shape recovery process. It was also found that after 10 cycles, the shape recovery performance had not degraded. When triggered by a voltage of 30 V, it took 72 seconds for the PGSE composite to recover its original shape (*R*_*r*_ = 97%). By increasing the triggering voltage to 50 V the recovery time decreased to 53 seconds.

## Conclusion

In this work we describe a simple and effective method to prepared PGSE composites. In the composite, graphite and AgNWs formed a 3D network, the AgNWs bridged the defects in RGO sheets and increased their conductivity due to their ability to behave as nanowires. The conductive network thus manufactured allowed the generation of joule heating thus achieving fast and uniform triggering of SMPs by electrical stimulus. The proposed PGSE composites with a volume ratio of AgNWs to graphite (1:1) demonstrate excellent conductivity and could be triggered within 60 seconds from the application of an electric field of approximately 0.8 V mm^−1^. The approach required only a standard electric power source and it is believed to be suitable for mass production. Moreover, the proposed method is eco-friendly, fast to manufacture, and low-cost when fabricating large-sized shape memory material. In the future, the composite material presented in this work may be a suitable candidate for the large-scale production of shape memory composite for versatile applications.

## Methods

### Synthesis of GO

GO was prepared by a modified Hummers method^45^. 23 mL of concentrated sulfuric acid was added into the mixture of 1 g graphite power and 0.5 g sodium nitrate in a 250 mL flask, and stirred for 15 min in ice bath. Subsequently, 3 g potassium permanganate was slowly added to the mixture under vigorous stirring and kept in ice bath for 30 min, and then the temperature increased to 35 °C followed by continuous stirring for 30 min. 117 mL deionized water was slowly added to the mixture. After that, 10 ml of H_2_O_2_ (30%) was added drop-wise. Finally the mixture was vacuum-filtrated and washed with 3% HCl and rinsed by deionized water several times until neutral pH of the solution was achieved.

### Synthesis of graphite/AgNWs hybrid foams

Vitamin C was purchased from Shanghai Lingfeng Chemical Reagent co., Ltd, China. The AgNWs were purchased from XFNANO co., Ltd. The commercial polyurethane (PU) foams were purchased from Sichuan Hongchang Plastics Industrial co., Ltd, China (brand name, “Maryya”). Foams were cleaned by several DI water rinses, followed by drying at 80 °C for 2 h. Subsequently, the clear PU foam was cut into thin strips of length × width × thickness equals to 50 mm × 20 mm × 4 mm. Each PU foam sample was immersed into a bottle containing a mixture of GO solution (7 mg mL^−1^), Vitamin C, and AgNWs (15 mg mL^−1^), and squeezed several times. The bottle was then put into a vacuum oven for 5 minutes under reduced pressure to remove air bubbles trapped in the foam. Subsequently, the bottle was closed and put in a convection oven where graphite was reduced by a green reductant-Vitamin C for four hours at 75 °C under atmospheric pressure^46^. With this process, GO could be reduced to graphite and self-assembled *in situ*. AgNWs were uniformly distributed in the graphite sheets. After reducing, the residual Vitamin C was removed by dialysis. At last, the product was dried to obtain the PGSs. PGSs with graphite to AgNWs corresponds to 1:2, 1:1 and 2:1 in volume ratio were synthesized in this way. After the removal of the polymer framework by pyrolysis of PGS under nitrogen atmosphere at 700 °C for 2 h, a 3D scaffold structure of graphite and AgNWs was obtained.

### Preparation of PGSE

Poly (ethylene glycol) diglycidyl ether (PEGGE) was purchased from Shanghai Rufa Chemical Technology. 4, 4′-Diaminodiphenylmethane (DDM) was purchased from Sinapharm Chemical Reagent co., Ltd, China. Jeffamine D-400 was purchased from Aladin Ltd. (Shanghai, China). All chemicals were used without additional purification. The composites were prepared by a vacuum infusion method. The graphite/AgNWs hybrid foam was placed in an aluminum foil mold slightly bigger than the aerogel. Subsequently the aerogel was immersed in a mixture of E51, PEGGE, DDM, and D-400 equals to a 9:1:1.58:0.79 weight ratio. D-400 and PEGGE are liquid at room temperature and could be used directly with no preheating. E51 and DDM were preheated at 105 °C and kept in liquid state. First, E51 and PEGGE were mixed by stirring and spreading evenly. Subsequently, the preheated mixture consisting of DDM and D-400 was added into the mixture previously described. The whole process was carried out at 105 °C. Then the graphite/AgNWs hybrid foam was put in vacuum oven for 5 minutes under reduced pressure to remove air bubbles trapped in the foam and to let the fluid diffuse into the hybrid foam. The composites were heated in an oven at 110 °C for 2 hours and post-cured at 150 °C for 5 hours. At last, both the sample’s ends were coated by conductive adhesive (mixture of Ag particles and epoxy resin) and two copper wires were fixed at each end.

### Measurement

The thickness of suspended graphite sheets were characterized by atomic force microscope (AFM, NanoScopeIIIa multimode AFM). The morphology and microstructure were characterized using a field emission scanning electron microscope (SEM, Hitachi, S-4800) with the acceleration voltage of 10 kV and transmission electron microscopy (TEM, JEOL JEM-2100F). X-ray photoelectron spectroscopic (XPS) measurements were performed on a Kratos AXIS Ultra DLD spectrometer with a monochromatic Al Ka X-ray source to verify the existence of chemical bonds before and after reduction. Electronic conductivities were measured using a four-point probes conductivity measurement device (RTS-8, PEOBES TECH, CHN). Thermo-gravimetric analysis (TGA, PerkinElmer, Pyris 1 TGA) was used to characterize the pyrolysis process of the samples, which was carried out with a heating rate of 10 °C min^−1^ from room temperature to 900 °C under a nitrogen atmosphere. Dynamic mechanical analysis (DMA) data were recorded on a DMA 8000 analyzer (PerkinElmer, USA). Measurements were done in the single cantilever bending mode. The temperature was restricted to a range between 0 °C to 100 °C. The temperature ramp rate was 3 °C min^−1^ and a frequency of 1 Hz was employed for all measurements. Shape recovery experiments were performed on an electric field of approximately 0.8 V mm^−1^ across the samples by means of a DC Power source (DH1765, DAHUA ELECTRONIC, CHN). The samples tested were heated in oven up to *T*_g_ + 25 °C at an approximate heating rate of 5 °C min^−1^ and held at temperature for 10 min for full heating. As the samples became malleable, they could be easily bent into a ‘U’ shape around a mandrel with a diameter of 10 mm at a bending rate of 10° s^−1^. Subsequently, the bent samples were fixed on the mandrel, immersed in cool water, and held for 10 min to ensure shape retention^47^. The shape recovery process was recorded by a camera and the recovery angles estimated with the help of a protractor. Finally, a thermal infrared imager (FLIR A615) was used to record the surface temperature distribution of the composite during the electrical actuation. The temperature range was set between 20 to 150 °C, and the reported accuracy of the infrared imager 0.05 °C.
